# Problematic Internet Use in High School Students in Guangdong Province, China

**DOI:** 10.1371/journal.pone.0019660

**Published:** 2011-05-06

**Authors:** Hui Wang, Xiaolan Zhou, Ciyong Lu, Jie Wu, Xueqing Deng, Lingyao Hong

**Affiliations:** Department of Medical Statistics and Epidemiology, School of Public Health, Sun Yat-sen University, Guangzhou, China; The University of Queensland, Australia

## Abstract

**Background:**

Problematic Internet Use (PIU) is a growing problem in Chinese adolescents. There are many risk factors for PIU, which are found at school and at home. This study was designed to investigate the prevalence of PIU and to investigate the potential risk factors for PIU among high school students in China.

**Methodology/Principal Findings:**

A cross-sectional study was conducted. A total of 14,296 high school students were surveyed in four cities in Guangdong province. Problematic Internet Use was assessed by the 20-item Young Internet Addiction Test (YIAT). Information was also collected on demographics, family and school-related factors and Internet usage patterns. Of the 14,296 students, 12,446 were Internet users. Of those, 12.2% (1,515) were identified as problematic Internet users (PIUs). Generalized mixed-model regression revealed that there was no gender difference between PIUs and non-PIUs. High study-related stress, having social friends, poor relations with teachers and students and conflictive family relationships were risk factors for PIU. Students who spent more time on-line were more likely to develop PIU. The habits of and purposes for Internet usage were diverse, influencing the susceptibility to PIU.

**Conclusions/Significance:**

PIU is common among high school students, and risk factors are found at home and at school. Teachers and parents should pay close attention to these risk factors. Effective measures are needed to prevent the spread of this problem.

## Introduction

Over the past few decades, the number of netizens in China has been increasing rapidly. According to the 24th China Internet development statistical report, as of 30 June 2009, there were 33.8 million people in China with access to the Internet. Of those, the group aged 10–29 years was the largest (62.8%) [1]. The average time spent on-line among adolescents was approximately 16.5 hours per week [2]. The Internet has now become an integral part of daily life; it is used for entertainment and communication as well as education. Despite its widely identified advantages, negative impacts of Internet use have progressively emerged, in particular, excessive use of the Internet. Since the mid-1990s, “Internet Addiction” has been proposed as a new type of addiction and mental health problem, similar to other established addictions such as alcoholism and compulsive gambling [3]. Young has described Internet addiction as an impulse-control disorder that does not involve an intoxicant [4]. Further studies utilized other methods to identify this disorder, which was also termed “problematic internet use” or “pathological internet use” [5]. Beard and Wolf defined problematic Internet use(PIU) as use of the Internet that creates psychological, social, school, and/or work difficulties in a person's life [6]. Indulging in the use of the Internet is associated with a variety of problems. Chou et al. reported that addicted subjects rated the impact of the Internet on their daily life, such as meals, sleep, and appointments, as significantly more negative than the non-addicted group [7]. In Tsai and Lin's study, Internet-dependent adolescents perceived that the Internet negatively affected their school performance and relationships with their parents [8]. PIU has become a serious problem.

Recently, many studies on PIU have been published. The majority of these focus on four topics. 1) How to assess PIU. Through on-line surveys and phone interviews, Young developed an eight-item Internet addiction diagnosis criteria which was a modification of the criteria for pathological gambling [4]. Based on the DSM-IV criteria and the observation of clinical case, Chen designed the Chinese Internet Addiction Scale containing 26 items in four dimensions: tolerance, withdrawal, compulsive behavior and other related factors [9]. Up until now, there had been no consensus on measurement instruments [10]. 2) The association between PIU and other problems. Ko found that after controlling for the effects of shared associated factors, adolescents with Internet addiction were more likely to display aggressive behaviors [11]. 3) Psychiatric features of adolescents with PIU. Yang reported that excessive Internet users scored significantly higher on anxiety, hostility and depression and they tended to be more lonely [12]. 4) Potential risk factors associated with PIU such as Internet usage patterns and socio-environmental factors. Although many studies have been carried out on this topic, some questions remain. First, some studies have recruited participants on-line or used a convenience sample [13], [14]. These studies have inherent biases, which make it difficult to accurately assess the prevalence of PIU as well as the relationship between influential factors and PIU. Second, many studies have been conducted among college students because they are deemed to be more vulnerable to Internet addiction than other groups [15], [16]. However, during adolescence, high school students usually experience dramatic changes in physiology and psychology, and may develop more serious problems than do individuals of other ages if they engage in problem behaviors. There is increasing evidence that PIU among high school students is emerging due to easy access to the Internet [17], [18]. Thus, high school students, like college students, are vulnerable to PIU.

For these reasons, we carried out a large-scale, cross-sectional study in Guangdong province. The main purpose of our study was to investigate the prevalence of PIU among high school students in China, and the relationship between PIU and potential factors. This study will contribute to our understanding of PIU among Chinese adolescents and help in designing educational policies to prevent problematic Internet use.

## Materials and Methods

### Study design and participants

A cross-sectional study was conducted to investigate the prevalence of PIU and to examine the relationship between potential influential factors and PIU. Participants were high school students recruited from four cities in Guangdong Province (Shenzhen, Guangzhou, Zhanjiang and Qingyuan). A stratified cluster random sampling was applied to choose participants. First, three key junior high schools, three regular junior high schools, two key senior high schools, two regular senior high schools and two vocational schools were selected in each city, and then two classes were selected from each grade of these schools. All students in the selected classes were invited to participate in this research. A total of 14,296 students were recruited to participant in the study. Of these, 1,850 did not use the Internet and the 12,446 who had Internet access provided usable information.

### Data collection

Self-completed questionnaires were distributed to all of the study participants on-site in their respective schools. The participants were requested to complete the questionnaire anonymously and the teachers were required to leave the classroom in order to minimize any potential information bias. The questionnaire consisted of three components: 1) Demographic information; 2) Family and school related factors; 3) Internet usage pattern. Demographic variables included age, gender, type of school and personal behavior. Family and school related factors included: (1) Family relations: please estimate the relationship between your family members. (2) Parental satisfaction: please estimate your parental' care. (3) Communication with parents: how often do you communicate with your parents? (4) Parents' education level: what are your parents' education levels? (5) Student's relationship with classmates and teachers: please estimate the relationship with your teachers and classmates. (6) Study-related stress: please estimate the stress coming from the study. All of these factors were self-rated. Internet usage pattern was assessed by examining the time spent on-line per day, the frequency of Internet use per week, and the purpose and location of Internet use. The Young's Internet Addiction Test (YIAT) was applied in order to assess problematic Internet use. The YIAT consists of 20 items. Each item is scored from 1 to 5, with 1 representing “not at all” and 5 representing “always”. Hence, possible total scores range from 20 to 100. The following cut-off points were applied to the total YIAT score 1) Normal Internet use: scores 20–49; 2) Potential problematic Internet use (PIUs): scores over 50 [19]. The split-half reliability was 0.859 and Cronbach's alpha was 0.902. Participants were fully informed of the purpose of the present study and were invited to participate voluntarily. Written consent letters were obtained from the school and students. All data were collected in November 2009. The study received approval from the Sun Yat-Sen University, School of Public Health Institutional Review Board.

### Statistical analysis

All statistical analyses were conducted using SPSS version 19.0. Descriptive analysis was used to describe the student's demographic characteristics and the prevalence of PIU. Chi-square tests were used to examine the difference between non-PIU and PIU. All factors that showed statistical significance in chi-square tests were further analyzed by multivariate analysis. We used generalized linear mixed-model regression in order to adjust for the school clustering effect. A statistical significance criterion of p<0.05 was applied for all the variables that remained in the final model.

## Results

### Prevalence of PIU

Of the 12,446 students who have ever used the Internet, 6,063 (48.7%) were male, and 6,383 (51.3%) were female. The mean age was 15.6, with a range from 10 to 23 years. Of the subjects, 22.8% (2,837) were from Qingyuan, 22.8% (2,838) were from Zhanjiang, 27.1% (3378) were from Chaozhou and 27.3% (3,393) were from Shenzhen. Among these, 10,931 (87.8%) were normal users, and 1515 (12.2%) met the criteria for PIU. Male students comprised 58.2% (882) of the problematic Internet users (PIUs). Of the subjects, 663 students reported smoking behaviors; 182 of these were PIUs. Some alcohol use was reported; 267 students drank more than four times in one month. Of those, 73 were PIUs. Other demographic characteristics and the distribution between PIUs and non-PIUs are shown in Table 1.

**Table 1:** Comparison of non-PIUs and PIUs over characteristic of the participants.

Variables	N of non-addicted (%)	N of addicted (%)	Total (%)	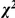
	(n = 10,931)	(n = 1,515)	(N = 12,446)	
Gender				62.357***
Males	5,181(47.4)	882(58.2)	6,063(48.7)	
Females	5,750(52.6)	633(41.8)	6,383(51.3)	
District				46.164***
Qingyuan	2,448(22.4)	389(25.7)	2,837(22.8)	
Zhanjiang	2,577(23.6)	261(17.2)	2,838(22.8)	
Huizhou	2,896(26.5)	482(31.8)	3,378(27.1)	
Shenzhen	3,010(27.5)	383(25.3)	3,393(27.3)	
Type of school				11.783*
Key senior high school	1,761(16.1)	259(17.1)	2,020(16.2)	
Regular senior high school	2,082(19.0)	303(20.0)	2,385(19.2)	
Vocational high school	1,785(16.3)	229(15.1)	2,014(16.2)	
Key junior high school	2,887(26.4)	351(23.2)	3,238(26.0)	
Regular junior high school	2,416(22.1)	373(24.6)	2,789(22.4)	
Smoking^a^				161.176***
No	10,312(94.3)	1,315(86.8)	11,627(93.4)	
Yes	478(4.4)	182(12.0)	663(5.3)	
Drinking/M				58.702***
<4 Times	10,737(98.2)	1,442(95.2)	12,179(97.9)	
≥4 Times	194(1.8)	73(4.8)	267(2.1)	
Social friends^b^				152.523***
No	5,891(53.9)	561(37.0)	6,452(51.8)	
Yes	5,017(45.9)	952(62.8)	5,969(48.0)	

*p<0.05,

**p<0.01,

***p<0.001.

Missing date exist in some categories.

a Smoking≥20 times in a half of a year.

b Adolescent who have dropped out of school.

### Family and school related factors and PIU

As shown in Table 2, without adjustment for other variables, PIU was significantly associated with a series of variables: family relations, parental satisfaction, communication with parents, study-related stress, financial situation, and relationships with classmates and teachers. There was no significant difference between the two groups with regard the mother's educational level 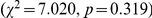 or the father's educational level 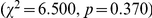 (data not shown in the Table).

**Table 2:** Comparison of non-PIUs and PIUs over family and school related factors.

Variables	N of non-addicted (%)	N of addicted (%)	Total (%)	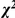
	(n = 10,931)	(n = 1,515)	(N = 12,446)	
Family relations				347.912***
Very supportive	3,704(33.9)	275(18.2)	3,979(32.0)	
Supportive	4,508(41.2)	592(39.1)	5,100(41.0)	
General	2,126(19.4)	441(29.1)	2,567(20.6)	
Conflictive	371(3.4)	113(7.5)	484(3.9)	
Very conflictive	139(1.3)	84(5.5)	223(1.8)	
Communication with parents			244.224***	
Very much	1,623(14.8)	149(9.8)	1,772(14.2)	
Much	3,270(29.9)	299(19.7)	3,569(28.7)	
Average	4,423(40.5)	638(42.1)	5,061(40.7)	
A little	1,388(12.7)	364(24.0)	1,752(14.1)	
None	94(0.9)	44(2.9)	138(1.1)	
Parental satisfaction				178.204***
Very satisfied	3,533(32.3)	303(20.0)	3,836(30.8)	
Satisfied	4,413(40.4)	577(38.1)	4,990(40.1)	
General	2,374(21.7)	493(32.5)	2,867(23.0)	
Dissatisfied	253(2.3)	76(5.0)	329(2.6)	
Very dissatisfied	272(2.5)	55(3.6)	327(2.6)	
Financial situation				27.117***
Very good	454(4.2)	44(2.9)	498(4.0)	
Good	2,526(23.1)	308(20.3)	2,834(22.8)	
General	6,493(59.4)	918(60.6)	7,411(59.5)	
Poor	1,163(10.6)	189(12.5)	1,352(10.9)	
Very poor	169(1.5)	43(2.8)	212(1.7)	
Study-related stress				191.078***
None	735(6.7)	133(8.8)	868(7.0)	
A little	1,476(13.5)	139(9.2)	1,615(13.0)	
Normal	4,766(43.6)	513(33.9)	5,279(42.4)	
Heavy	3,197(29.2)	506(33.4)	3,703(29.8)	
Very heavy	649(5.9)	213(14.1)	862(6.9)	
Classmate relations				183.010***
Very good	3,637(33.3)	368(24.3)	4,005(32.2)	
Good	5,104(46.7)	646(42.6)	5,750(46.2)	
General	1,961(17.9)	423(27.9)	2,384(19.2)	
Poor	105(1.0)	49(3.2)	154(1.2)	
Very Poor	46(0.4)	20(1.3)	66(0.5)	
Teacher relations				277.250***
Very good	2,002(18.3)	145(9.6)	2,147(17.3)	
Good	4,409(40.3)	481(31.7)	4,890(39.3)	
General	4,125(37.7)	743(49.0)	4,868(39.1)	
Poor	235(2.1)	88(5.8)	323(2.6)	
Very poor	82(0.8)	50(3.3)	132(1.1)	

*p<0.05,

**p<0.01,

***p<0.001.

Missing date exist in some categories.

### Internet usage and PIU

The most common use of the Internet was for entertainment (n = 8,637, 69.4%), followed by communication with classmates (n = 7,815, 62.8%) and learning (n = 6027, 48.4%). Most students (72.7%) reported using the Internet at home. Approximately 9.9% of PIUs spent more than 8 hours per day on the Internet, while only 2.1% of the non-PIUs spent more than 8 hours per day using the Internet. Of the non-PIUs, 4.7% non-PIUs spent 4–6 hours per day on the Internet, compared with 11.2% among PIUs. The Chi-square test revealed significant differences between the two groups (p<0.005) (See Table 3).

**Table 3:** Comparison of non-PIUs and PIUs over history of Internet usage.

Variables	N of non-addicted (%)	N of addicted (%)	Total (%)	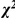
	(n = 10,931)	(n = 1,515)	(N = 12,446)	
Average numbers of hours online/Day				679.483***
<2 h/D	7,667(70.1)	657(43.4)	8,324(66.9)	
2–4 h/D	2,379(21.8)	455(30.0)	2,834(22.8)	
4–6 h/D	509(4.7)	169(11.2)	678(5.4)	
6–8 h/D	151(1.4)	84(5.5)	235(1.9)	
>8 h/D	225(2.1)	150(9.9)	375(3.0)	
Frequency of weekly Internet use				603.332***
<2 times/W	5,774(52.8)	425(28.1)	6,199(49.8)	
3–4 times/W	3,290(30.1)	498(32.9)	3,788(30.4)	
5–6 times/W	885(8.1)	196(12.9)	1,081(8.7)	
7–8 times/W	483(4.4)	137(9.0)	620(5.0)	
>9 times/W	499(4.6)	259(17.1)	758(6.1)	
Online sites				92.609***
Internet cafe	1,232(11.3)	256(16.9)	1,488(12.0)	
Home	7,932(72.6)	1,122(74.1)	9,054(72.7)	
School	117(1.1)	4(0.3)	121(1.0)	
Relative/friends' home	469(4.3)	16(1.1)	485(3.9)	
Mobile	1,092(10.0)	106(7.0)	1,198(9.6)	
others	53(0.5)	8(0.5)	61(0.5)	
Entertainment				106.544***
Yes	7,410(67.8)	1,227(81.0)	8,637(69.4)	
No	3,412(31.2)	279(18.4)	3,691(29.7)	
Information gathering				437.372***
Yes	5,674(51.9)	353(23.3)	6,027(48.4)	
No	5,095(46.6)	1,133(74.8)	6,228(50.0)	
Making friends				59.578***
Yes	1,649(15.1)	350(23.1)	1,999(16.1)	
No	8,951(81.9)	1,140(75.2)	10,091(81.1)	
Communicating with friends				112.738***
Yes	7051(64.5)	764(50.4)	7815(62.8)	
No	3756(34.4)	730(48.2)	4,486(36.0)	
Others				1.034
Yes	522(4.8)	64(4.2)	586(4.7)	
No	10,064(92.1	1,416(93.5)	11,480(92.9)	

*p<0.05,

**p<0.01,

***p<0.001.

Missing date exist in some categories.

### Multivariate analyses for PIU

The results of the generalized mixed-model regression are presented in Table 4. They suggest that PIUs are more likely to experience study-related stress and poor relationships with teachers and classmates. Conflictive family relations and poor financial situation are associated with a higher probability of PIUs who use the Internet mainly for entertainment. In addition, those who use the Internet at Internet cafes were more likely to develop PIU.

**Table 4:** Generalized linear mixed-model for risk factors of problematic Internet use.

Variables	Adjusted OR	95%CI for Adjusted OR	Variables	Adjusted OR	95%CI for Adjusted OR
Social friends (No vs. Yes)	1.46	1.27–1.69**	Parental satisfaction*		
Gender(Male vs. Female)	1.02	0.95–1.25	Very satisfied	1	
Smoke (Yes vs. No)	1.15	0.91–1.46	Satisfied	1.78	1.08–2.94**
Drink (Yes vs. No)	1.22	0.88–1.77	General	1.42	0.93–2.16
Zone			Dissatisfied	1.53	1.00–2.32
Qingyuan	1		Very dissatisfied	1.29	0.83–1.99
Zhanjiang	0.72	0.55–0.95**	Classmate relations*		
Chaozhou	1.02	0.81–1.28	Very good	1	
Shenzhen	0.91	0.72–1.16	Good	1.1	0.93–1.31
Type of school*			General	1.45	1.17–1.78**
Key senior high school	1		Poor	2.42	1.55–3.77**
Regular senior high school	1.22	0.68–1.14	Very Poor	1.88	0.89–4.00
Vocational high school	0.88	0.36–0.66**	Teacher relations*		
Key junior high school	0.48	0.72–1.18	Very good	1	
Regular junior high school	0.91	0.76–1.27	Good	1.11	0.88–1.41
Study-related stress*			General	1.18	0.93–1.52
None	1		Poor	1.49	1.02–2.17**
A little	1.02	0.75–1.38	Very poor	1.47	0.88–2.45
Normal	1.09	0.84–1.41	Numbers of hours online/D*		
Heavy	1.59	1.22–2.07**	<2 h/D	1	
Very heavy	2.19	1.62–2.96**	2–4 h/D	1.56	1.34–1.82**
Family relations *			4–6 h/D	2.13	1.68–2.71**
Very supportive	1		6–8 h/D	3.6	2.58–5.02**
Supportive	1.42	1.18–1.72**	>8 h/D	3.01	2.25–4.04**
General	1.68	1.34–2.10**	Frequency of weekly Internet use/W*		
Conflictive	2.01	1.45–2.80**	<2 times/W	1	
Very conflictive	2.60	1.70–3.98**	3–4 times/W	1.7	1.45–1.99**
Financial situation*			5–6 times/W	2.07	1.66–2.59**
Very good	1		7–8 times/W	2.38	1.83–3.08**
Good	1.39	0.95–2.05	>9 times/W	2.74	2.15–3.49**
General	1.52	1.04–2.20**	On-line sites*		
Poor	1.77	1.17–2.69**	Internet cafe	1	
Very Poor	1.37	0.77–2.42	Home	0.94	0.76–1.15
Communication with parents*			School	0.43	0.15–1.22
Very much	1		Relative or friends' home	0.33	0.19–0.57**
Much	0.94	0.57–1.53	Mobile	0.54	0.40–0.72**
Average	0.78	0.47–1.27	Others	0.75	0.32–1.79
A little	0.75	0.45–1.24	Entertainment (No vs. Yes)	1.68	1.42–1.97**
None	1.09	0.64–1.84	Learning (No vs. Yes)	0.42	0.37–0.49**
Communication with friends(No vs. Yes)	0.82	0.72–0.93**	Making friends(No vs. Yes)	1.54	1.32–1.80**

*:using the first category as reference;

**p<0.05.

## Discussion

### Prevalence of PIU

To the best of our knowledge, this investigation of 14,296 Chinese high school students is the largest cross-sectional study of high school students undertaken to date. The information provided here may help us better understand the factors that are associated with PIU. In this survey, the prevalence of PIU was 12.2% (1515). Similar research has been performed by others. Lam and colleagues carried out a study among high school students using Young's 20-item IAT. They reported that 10.8% (168) were diagnosed as Internet addicted users, similar to our study [20]. In Luca's study, 98 adolescents surveyed with Young's 20-item test found a PIU prevalence of 36.7%, which was higher than our study. This may due to smaller sample size [21]. Using the 20-item YIAT, Ni and colleagues identified 6.44% of 3,557 first-year university students as Internet addicted [22], which was lower than our study. These results suggest that PIU may be more severe among high school students in China. Similar studies were also carried out that utilized different scales. F. Cao and L. Su reported that the incidence rate of Internet addiction among 2,620 high school students in Changsha city was 2.4%, which was identified by using a modified version of the YDQ criteria [23]. In other countries, the rate of Internet addiction among adolescents varies widely, from 3.8% to 36.7% [18], [21]. Thus, the comparison of prevalence data is complicated due to the diversity of assessment tools applied and to different samples and social contexts.

Previous studies identified gender as a risk factor for PIU [20], [24]. However, Kim suggested that the different distribution of Internet addiction between males and females might be attributable to the different on-line activities of males and females [25]. Males tend use the Internet for entertainment, such as on-line gaming and Internet gambling, which are both associated with compulsive Internet use. Hall argued that the changes in the availability and the nature of Internet service have eliminated the gender gap in the Internet-addicted students [26]. Khazaal also did not find a significant relationship between YIAT score and gender [19]. Our results are in agreement with Khazaal. In the multivariate analyse, after adjusting for the different use modalities of the Internet, gender was not a risk factor. For this reason, females should not be ignored in PIU prevention programs.

Having social friends was another influential factor for PIU. Our results showed that students who had friends who dropped out of school were almost 1.5 times more likely to demonstrate PIU than those whose friends did not drop out (OR = 1.46, 95% CI = 1.27–1.69). This result may be ascribed to peer effect. Adolescents who drop out of school tend to spend more time on the Internet. Students in contact with those people easily engaged in excessive Internet use in this context. Much research has been conducted to explore the effect of peer influence on problem behaviors. For instance, according to Norton and Lindrooth, peer smoking has a strong positive effect on smoking in adolescents [27]. We assumed that peer effects might be a risk factor for PIU. However, studies on the effect of peer influence on PIU are rare, and further research is needed on this topic.

In our study, there was not an association between alcohol and tobacco use in the final model (p>0.05), consistent with other studies [28]. It has been suggested that those problematic behaviors share similar risk factors, such as poor intra-family relationships. After controlling for the potential family related factors in the multiple regression models, the association disappeared.

### Family and school related factors and PIU

Family plays a very important role in the psychosocial development and well being of children. Problem behaviors are more likely if families have high levels of conflict. Yen et al. reported that high parent-adolescent conflict predicted Internet addiction in adolescents. Adolescents with a higher conflict level with their parents refused to obey the supervision of their parents, including the rules set for Internet use [28]. The present study found similar results; conflictive family relations are a risk factor for PIU, increasing the OR over one time (OR = 2.01, 95%CI = 1.45–2.80; OR = 2.60, 95%CI = 1.70–3.98). Families with high levels of conflict were less likely to have high levels of parent-child involvement and adequate parental monitoring [29], which would predict adolescents being predisposed to problematic internet use. Other family factors such as family communication, parental satisfaction were correlated with PIU by Chi-square tests, but after adjustment for family relations, these correlations disappeared. We presumed that the correlations showed in the univariate analyses resulted from the relationship between the family relations and PIU. Contrary to previous reports, we failed to find an association or tendency between PIU and the parental educational level. This result suggests to us that most parents realize the problems or negative effects that adolescents may suffer in using Internet, so parents urge children to make the best use of the Internet, going so far as to monitor and restrict improper Internet use. As long as the parents continued to exercise loving care and control over them, students with parents with low educational levels did not have a higher probability for PIU.

With regard to school-related factors, we found that students with study-related stress and poor classmate relationships had a higher probability of PIU, consistent with past research. Luca's study suggested that a low quality of interpersonal relationships can expose adolescents to an increased risk of developing PIU [21]. The Internet provides a place for users to escape from reality and seek acceptance. A study of 700 college students found that most stressful events, including academic stress, social communication and other life stressors were more frequent in the PIU group than in the non-PIU group [30]. Another study found that cumulative stress significantly increased the risk for PIU [31]. From these results, it can be inferred that a high dependency on Internet use provided subjects with an alternative to real-life relationships that are associated with a lack of interpersonal skills.

### Internet usage pattern and PIU

We found that problematic Internet users spent more time on the Internet and used the Internet more frequently per week than non-PIUs. Those who spent more than 8 hours a day on-line had a higher probability of developing PIU than those who spent less than 2 hours a day on-line (OR = 3.01, 95%CI = 2.25–4.04). A relationship between hours spent on-line and PIU has been reported in several studies. In Sunny's study, dependents spent an average of 28.1 hours on-line per week compared to non-dependents, who spent about 12.1 hours per week. The difference between dependent and non-dependent users was significant (t = 8.868, *p*<0.001) [32]. Similarly, Chou reported that non-addicts spent about 5–10 hours on-line per week, while non-addicts spent 20–25 hours on-line per week. He postulated that Internet addicted users have to spend increasing amounts of time on the Internet to achieve the desired effect [33]. Therefore, restricting adolescents' time on-line would be an effective measure to prevent PIU.

In our study, most PIUs used the Internet for entertainment. We found that using the Internet for entertainment was a powerful predictor for PIU (OR = 1.68, 95% CI = 1.42–1.97). The second powerful predictor was making friends (OR = 1.54, 95% CI = 1.32–1.80). We presume that problematic Internet users are more likely to use the interactive functions of the Internet, such as on-line games and chatting, which might satisfy user's needs and actually facilitate pathological use [34]. Similar studies have been carried out. Huang reported that 55.9% of problematic Internet users used the Internet for gaming, compared with 33.19% of non-problematic users (P<0.05) [35]. In Sherk and College's study, playing on-line games was a powerful predicator of Internet addiction, increasing the odds ratio by 70% (OR = 1.70, 95% CI = 1.46–1.90) [36]. According to our results, those using the Internet for communication with friends were less likely to develop PIU (OR = 0.41, 95% CI = 0.36–0.47). This finding is in line with previous studies. Students in Taiwan reported that they generally experienced positive effects by using the Internet for communication. The Internet can be used to maintain meaningful interpersonal relationships [37]. Kraut et al. proposed a “rich get richer” model, suggesting that the Internet provided more benefits to those who were already well adjusted [38].

The site of Internet usage was also related to PIU. Internet users primarily chose their own home as the location for surfing on-line; Internet cafes were second on the list. The generalized linear mixed-model revealed that compared with other on-line sites, students choosing Internet cafes had a higher OR for PIU than other sites, for example at relatives or friends homes. It is important to note that both locations allow adolescents to freely surf the Internet without the pressures of authority or parental control [24]. Internet cafes not only provide the virtual interaction of personal relationships but also the social support that was the real interaction among people [39]. In the Internet cafe, students can seek acceptance and support from members of a social network and alleviate guilt, as well as find satisfaction in life.

Our results should be interpreted in light of several limitations. First, the cross-sectional research design of the present study could not confirm causal relationships between PIU and possible influential factors. Second, we were lacking information from the parents; assessment of family-related factors was based solely on self-report data. Third, not all possible factors were included in our study. Further studies should attempt to determine additional predictive factors by identifying the causal relationship between PIU and the psychological characteristics of adolescents.

In conclusion, adolescence is a time in which people experience significant biological, psychological, and social changes. Those who have trouble navigating these developmental challenges are particularly vulnerable to PIU. Although our study is preliminary and there may be many relevant factors that were neglected, 12.1% of the high school students surveyed exhibited PIU. In addition to family and school related factors, other influential factors including Internet usage patterns are associated with PIU. Special attention should be paid to those high school students who display these risk factors. Further research is needed to understand the underlying mechanisms that affect PIU and to explore effective preventative treatment strategies.
